# Impact of polymorphisms in *CYP* and *UGT* enzymes and *ABC* and *SLCO1B1* transporters on the pharmacokinetics and safety of desvenlafaxine

**DOI:** 10.3389/fphar.2023.1110460

**Published:** 2023-02-02

**Authors:** Sofía Calleja, Pablo Zubiaur, Dolores Ochoa, Gonzalo Villapalos-García, Gina Mejia-Abril, Paula Soria-Chacartegui, Marcos Navares-Gómez, Alejandro de Miguel, Manuel Román, Samuel Martín-Vílchez, Francisco Abad-Santos

**Affiliations:** ^1^ Clinical Pharmacology Department, Hospital Universitario de La Princesa, Instituto Teófilo Hernando, Instituto de Investigación Sanitaria La Princesa (IP), Universidad Autónoma de Madrid (UAM), Madrid, Spain; ^2^ Division of Clinical Pharmacology, Toxicology and Therapeutic Innovation, Children’s Mercy Research Institute, Kansas City, MO, United States; ^3^ Centro de Investigación Biomédica en Red de Enfermedades Hepáticas y Digestivas (CIBERehd), Instituto de Salud Carlos III, Madrid, Spain

**Keywords:** desvenlafaxine, pharmacogenetics, major depressive disorder, precision medicine, pharmacokinetics, safety

## Abstract

Venlafaxine pharmacokinetic variability and pharmacotherapy outcomes are well known to be related to CYP2D6 pharmacogenetic phenotype. In contrast, scarce pharmacogenetic information is available nowadays concerning desvenlafaxine, its active metabolite first marketed in 2012. The aim of this study was to evaluate the impact of 29 alleles in 12 candidate genes (e.g., *CYP* enzymes like *CYP2D6*, *CYP3A4*, or *CYP2C19*; *ABC* transporters like *ABCB1*; *SLCO1B1*; and *UGT* enzymes like *UGT1A1*) on desvenlafaxine pharmacokinetic variability and tolerability. Pharmacokinetic parameters and adverse drug reaction (ADR) incidence obtained from six bioequivalence clinical trials (*n* = 98) evaluating desvenlafaxine formulations (five with single dose administration and one with multiple-dose administration) were analyzed. No genetic polymorphism was related to pharmacokinetic variability or ADR incidence. Volunteers enrolled in the multiple-dose clinical trial also showed a higher incidence of ADRs, e.g., xerostomia or appetite disorders. Volunteers experiencing any ADR showed a significantly higher area under the time-concentration curve (AUC) than those not experiencing any ADR (5115.35 vs. 4279.04 ng*h/mL, respectively, *p* = 0.034). In conclusion, the strong dose-dependent relationship with the occurrence of ADRs confirms that the mechanism of action of desvenlafaxine is essentially dose-dependent.

## Introduction

Venlafaxine belongs to the group serotonin-noradrenaline re-uptake inhibitor (SNRI) antidepressant drugs. It was first marketed in 1995 and is widely used in the management of depression. Venlafaxine is metabolized by CYP2D6 resulting in its active metabolite desmethylvenlafaxine (i.e. desvenlafaxine), which was marketed in 2012 with the aim of reducing venlafaxine’s side effects (Food and Drug Administration, 2011). Desvenlafaxine daily dose ranges between 50 and 100 mg. All the pharmaceutical forms marketed are prolonged-release tablets. After oral administration, desvenlafaxine is highly absorbed, with a bioavailability of 80%. Maximum plasma concentrations (C_max_) are observed 7.5 h after oral administration (t_max_) (Liebowitz and Tourian, 2010). The area under the time-concentration curve (AUC) in a 24 h dosing interval at steady state (AUC_τ_) with the 100 mg dose is 6747 ng*h/mL, and the C_max_ is 376 ng/mL. The binding of desvenlafaxine to plasma proteins is low (30%) and the mean terminal half-life (t_1/2_), is approximately 11 h (Spanish Drug Agency, 2012). It is mostly metabolized by O-glucuronide conjugation by UDP-glucuronyltransferase (UGT) enzymes, including UGT1A1, UGT1A3, UGT2B4, UGT2B15, and UGT2B17. To a lesser extent, it undergoes oxidative metabolism by the cytochrome p450 (CYP) isoform CYP3A4 (Sproule et al., 2008; Preskorn et al., 2009; DrugBank, 2010). Venlafaxine structure contains a tertiary amine and a methoxy group, which can be N- and O-demethylated, respectively. The N-demethylation to N-desmethyl venlafaxine (NDV) is catalyzed by CYP3A4 and CYP2C19, while the O-demethylation to desvenlafaxine is catalyzed by CYP2D6 and CYP2C19 (Fogelman, 1999). When desvenlafaxine is administered instead of venlafaxine, the N-demethylation to N, O-didesmethyl venlafaxine is assumed to be mediated by means of CYP3A4 and CYP2C19 (pathway available at: https://www.pharmgkb.org/pathway/PA166014758).

*CYP2D6* polymorphism conditions venlafaxine exposure and response. In fact, the Royal Dutch Pharmacist’s Association (KNMP) Pharmacogenomics Working Group (DPWG) includes the *CYP2D6*-venlafaxine drug-gene pair in their recommendations list. Briefly, an alternative drug that is not metabolized by CYP2D6 or a dose reduction should be considered for CYP2D6 intermediate metabolizers (IMs) and poor metabolizers (PMs) and a 150% dose increased should be considered for ultrarapid metabolizers (UMs) if necessary (Nichols et al., 2009; Nichols et al., 2013; Dutch Pharmacogenetics Working Group, 2022). In contrast, desvenlafaxine is apparently not affected by CYP2D6 phenotype and scarce pharmacogenetic information is available nowadays for this drug. One study evaluated the impact of *ABCB1* rs1045642, *ABCC1* rs212090, and *UGT1A1* rs8175347 on desvenlafaxine dose requirements (Bousman et al., 2017). However, several additional pharmacogenetic biomarkers could be relevant concerning desvenlafaxine pharmacokinetics and/or dose requirements. Hence, the aim of this study was to evaluate the impact of 29 alleles in 12 candidate genes (i.e., *ABCB1*, *ABCG2*, *CYP2B6*, *CYP2C19*, *CYP2C8*, *CYP2C9*, *CYP2D6*, *CYP3A4*, *CYP3A5*, *SLCO1B1*, *UGT1A1*, and *UGT2B15*) on desvenlafaxine pharmacokinetic variability and tolerability.

## Materials and methods

### Study population

The study population comprised 98 healthy volunteers enrolled in six different desvenlafaxine bioequivalence clinical trials performed at the Clinical Trial Unit of Hospital Universitario de La Princesa (UECHUP). All subjects included in the study signed two written informed consent forms, one for their participation in the clinical trial and another one for pharmacogenetic studies. They were free to withdraw from the study at any time. Study protocols were revised and approved by the Hospital’s Research Ethics Committee and by the Spanish Drugs Agency (AEMPS). Complying with Spanish and European legislation on research in humans, all of them were accomplished under the Good Clinical Practice guidelines and endorsing the Declaration of Helsinki. EudraCT numbers for clinical trials A to F were as follows: 2019-000628-17, 2019-002739-26, 2019-003272-39, 2019-004289-16, 2019-004882-41, and 2020-003002-31, respectively.

Inclusion criteria were as follows: age 18-55 years, being free from any psychiatric or organic conditions, having normal vital signs and electrocardiogram (ECG), normal medical records and physical examination and no clinically significant abnormalities in serology, hematology, coagulation, biochemistry, and urinalysis. Exclusion criteria comprised the following: presenting clinically significant biochemical alterations, having received pharmacological treatment in the last 15 days or any kind of medication in the 48 h prior to receiving the study drug, a body mass index (BMI) outside the 18.5–30 kg/m^2^ range, having donated blood in the previous month, history of sensitivity to any drug, suspected consumption of controlled substances, smokers, daily consumers of alcohol and/or acute alcohol poisoning in the last week, pregnant or breastfeeding women, having participated in a similar study within the previous 3 months and swallowing difficulty.

### Study design and procedures

The current observational pharmacogenetic study was based on six independent phase I bioequivalence clinical trials (A, B, C, D, E, F). In three of them (A, E, F), two desvenlafaxine 100 mg prolonged release tablets formulations (one test formulation, T and one reference formulation, R) were administered once (single-dose); in two of them (B, D) two desvenlafaxine 50 mg prolonged release tablets formulations (T and R) were administered once (single-dose); in one of them (C), two desvenlafaxine 100 mg prolonged release tablets formulations (T and R) were administered several times (100 mg daily for 6 days), and the calculations to demonstrate bioequivalence were performed in steady-state. All of them were open-label, crossover, and randomized clinical trials, two sequence, two periods, with a wash-out period of at least 7 days (except for C clinical trial, without washout period between each period). The volunteers were hospitalized from 10 h before to 24 h after dosing in both periods. Formulations were administered by oral route under fasting conditions (A, B, C, E) or fed conditions (D, F) with 240 mL of water. Only the reference formulations (Pristiq^®^ 50 or 100 mg prolonged release tablets, Pfizer, Spain) for each volunteer in each clinical trial were considered for this study.

Blood samples were collected in 3 mL EDTA-K_2_ tubes at several-points between predose and 72 h after dosing (A, B, D, E, F) or 24 h after dosing (C) (i.e., during an administration interval, τ). After the extraction (direct venipuncture or from an indwelling cannula), the tube was put into an ice-water bath. The tubes were centrifuged at 4°C during 10 min at 1900 G. Once centrifuged, 0.5 mL of plasma was aliquoted and stored in a freezer at −20°C ± 5°C, until its shipment to an external analytical laboratory. The analytical method involved a protein precipitation extraction procedure with 0.1% formic acid in methanol, and subsequent reversed phase high performance liquid chromatography coupled to a tandem mass spectrometry (HPLC-MS/MS), with a lower limit of quantification (LLOQ) of 1 ng/mL.

### Pharmacokinetics analysis

Pharmacokinetic parameters were estimated by non-compartmental methods using WinNonLin Professional Edition (version 8.1 for A, B, D, and E and version 8.3 for C and F, Scientific Consulting, Inc., Cary United States) pharmacokinetic program. Two pharmacokinetic parameters were obtained directly from raw data: t_max_ and C_max_ (C_max-ss_ in steady state for study C). The rest of the parameters are obtained by the trapezoidal rule or by non-compartmental methods. The AUC from zero to the last observed concentration time (t) (AUC_t_) and AUC_Ƭ_ were calculated using the trapezoidal rule. The AUC from t time to infinity (AUC_t→inf_) was calculated dividing the concentration at t time (C_t_) by the elimination constant (K_e_), obtained by linear regression of the log-linear phase of the plasma level curve. The AUC from administration to infinity (AUC_∞_) was obtained adding AUC_t_ to AUC_t→inf_. The t_1/2_ we calculated as -ln2/k_e_.

### Safety

The safety assessment consisted of the evaluation of abnormalities in analytical values, blood (biochemistry, coagulation, haematology, serology) and urine parameters (urianalysis, pregnancy test, abuse drugs, and cotinine test), physical examination, vital signs (blood pressure and heart rate), serial ECG or any other clinically relevant event. Adverse events (AEs) spontaneously reported by volunteers or reported after open question were also collected. Causality was determined using the Spanish Pharmacovigilance System algorithm (Aguirre and García, 2016), according to five types of AE: definite, probable, possible, unlikely and unrelated. Only definite, probable, or possible AEs were considered as adverse drug reactions (ADRs) and included in the statistical analysis.

### Genotyping, haplotyping, and phenotyping

One EDTA-K_2_ tube used for pharmacokinetic profiling was reused for genotyping. After plasma separation, the cell concentrate was resuspended in NaCl 0.9% for the preservation of the blood. A Maxwell^®^ RSC Instrument (Promega, United States) was used for the extraction of DNA. For the genotyping, a QuantStudio 12 k flex (ThermoFisher, United States) was used with two thermal blocks: the OpenArray for the genotyping of 24 out of 27 variants and the 96-well fast thermal-block for CNV determination and the genotyping of the remaining 3 (Zubiaur et al., 2020). Therefore, 27 variants in 12 genes were included in the present work, which allowed de inference of 29 alleles (see Table 1). Genes were selected based on their known involvement in desvenlafaxine pharmacokinetics (e.g., *CYP2C19*, *ABCB1*, or *CYP3A4*) or for their potential involvement with a more exploratory purpose (e.g., other relevant CYPs like *CYP2D6* or *CYP3A* isoforms like *CYP3A5*, other transporters like *ABCG2* and *SLCO1B1* or other *CYP2C* enzymes like *CYP2C19* or *CYP2C9*). Variants in potential candidate genes like *UGT1A3* or *UGT2B4* were ruled out as no variant is demonstrated to impact the enzyme’s function to date. A copy number variation (CNV) assay, which allows the identification of the gene deletion (*5) or gene duplications (xN), was performed for *CYP2D6* as described previously (Belmonte et al., 2018). The following SNPs were manually genotyped with TaqMan assays following the manufacturer (ThermoFisher) recommendations: *ABCG2* rs2231142, *CYP3A4* rs67666821, and *UGT2B15* rs1902023 (Table 1).

**TABLE 1:** Genes, alleles, and variants genotyped and analyzed.

Gene	Allele	Variant
*ABCB1*	Legacy name: C1236T	rs1128503
	Legacy name: C3435T	rs1045642
	Legacy name: G2677 T/A	rs2032582
*ABCG2*	N/A	rs2231142
*CYP2B6*	*4	rs2279343
	*5	rs3211371
	*6	rs3745274, rs2279343
	*7	rs3745274, rs2279343, rs3211371
	*9	rs3745274
*CYP2C19*	*2	rs4244285
	*3	rs4986893
	*17	rs12248560
*CYP2C8*	*2	rs11572103
	*3	rs10509681
	*4	rs1058930
*CYP2C9*	*2	rs1799853
	*3	rs1057910
*CYP2D6*	*4	rs3892097
	*6	rs5030655
	*9	rs5030656
	*10	rs1065852
	*41	rs28371725
*CYP3A4*	*2	rs55785340
	*20	rs67666821
	*22	rs35599367
*CYP3A5*	*3	rs776746
*SLCOB1*	*5	rs4149056
*UGT1A1*	*80	rs887829
*UGT2B15*	N/A	rs1902023

*UGT1A1* rs887829 (*80) was used as a surrogate biomarker for *28. The tagSNP for CYP2D6*10 (rs1065852) also appears in *4 alleles; a *10 allele call is only possible when rs3892097 is not present. The same principle is applied for *CYP2B6* alleles. A copy number variation (CNV) assay was performed for *CYP2D6* to detect the gene deletion (*5) and duplications (xN).

Clinical Pharmacogenetics Implementation (CPIC) or DPWG pharmacogenetic guidelines were used to infer pharmacogenetic phenotypes when available: *CYP2B6* (Desta et al., 2019)*, CYP2C19* (Hicks et al., 2015; Lee et al., 2022), *CYP2C9* (Theken et al., 2020; Cooper-DeHoff et al., 2022), *CYP2D6* (Hicks et al., 2015; Brown et al., 2019), *CYP3A4* (Dutch Pharmacogenetics Working Group, 2021a), *CYP3A5* (Birdwell et al., 2015), *SLCOB1* (Cooper-DeHoff et al., 2022), *UGT1A1* (Gammal et al., 2016; Dutch Pharmacogenetics Working Group, 2021b)*, ABCG2* (Cooper-DeHoff et al., 2022). *ABCB1*, *CYP2C8*, and *UGT2B15* variants were individually analyzed as the pharmacogenetic phenotype has not been optimized to date.

### Statistical analysis

The SPSS software was used for statistical analysis (version 26.0, SPSS Inc., Chicago, United States). Firstly, a univariate analysis was performed. For the comparison of means, a *t*-test (variables with two categories) or an ANOVA test followed by a Bonferroni *post hoc* (variables with three or more categories) was accomplished. For the comparison of the incidence of ADRs according to categorical variables, a Chi-squared or a Fisher exact test were used to infer statistical significance in contingence tables. Afterwards, each pharmacokinetic parameter or ADR were individually analyzed with a multivariate analysis by means of linear regression (pharmacokinetic parameters) or logistic regression (ADRs). Categorical variables with more than two categories, such as polymorphisms, were transformed to *dummy* variables. Moreover, any phenotype or genotype with *p* < 0.05 in the univariate analysis as well as race and sex (covariates) were included. Pharmacokinetic variables were logarithmically transformed for statistical inference to ensure a normal distribution. AUC and C_max_ were divided by the dose/weight (DW) ratio to correct the impact of dose and weight on drug exposure. For the evaluation of ADRs, pharmacokinetic parameters without DW correction were included as independent variables in the logistic regression. The Bonferroni correction for multiple comparisons was applied.

## Results

A total of 98 healthy volunteers participated in the pharmacogenetic study. Mean age was 31.0 ± 8.9 years old, with evenly distributed proportions of males (*n* = 52) and females (*n* = 46) with 26 Caucasians and 72 healthy volunteers with Mixed race (including 71 Latin-Americans and one Black) (Table 2). Females exhibited lower height (*p* < 0.001) than males and lower weight (*p* < 0.001). In addition, the body mass index (BMI) of Caucasians was significantly lower than that of Mixed-race healthy volunteers (*p* = 0.010) (Table 2).

**TABLE 2:** Demographic characteristics of the study population according to sex, race, and clinical trial.

Variable		N	Age (years)		Height (m)		Weight (kg)		BMI (kg/m^2^)	
			Mean	SD	Mean	SD	Mean	SD	Mean	SD
Sex	Female	46	32.02	9.88	1.62	0.05	62.95	7.8	24.07	2.41
	Male	52	30.1	7.86	1.75*	**0.07**	75.34*	**10.68**	24.59	2.64
Race	Caucasian	26	29.27	9.36	1.71	0.11	68.02	12.6	23.26	2.70
	Mixed	72	31.63	8.67	1.68	0.08	70.07	10.78	24.74*	**2.37**
Clinical trial	A	18	29.50	8.33	1.68	0.09	67.62	7.94	24.08	2.86
	B	29	29.86	6.61	1.69	0.09	69.44	9.86	24.33	2.24
	C	9	34.67	12.41	1.67	0.08	70.81	14.25	25.28	3.56
	D	19	32.05	8.92	1.71	0.09	69.67	11.89	23.75	2.16
	E	17	32.65	11.35	1.68	0.10	70.31	13.39	24.64	2.66
	F	6	27.5	5.72	1.68	0.12	71.00	16.52	24.88	2.16
Total		98	31.00	8.87	1.69	0.09	69.52	11.26	24.35	2.53

*p< 0.05 after *t*-test.

### Pharmacokinetics

The total AUC_∞_ and C_max_ were 4594.79 ± 1871.71 ng*h/mL and 187.19 ± 85.62 ng/mL respectively. Uncorrected mean (SD) AUC_∞_ and C_max_ values are shown in Table 3 according to the clinical trial design.

**TABLE 3:** Uncorrected AUC and C_max_ values according to the study design.

Clinical Trial design		AUC (ng*h/mL)		C_max_ (ng/mL)	
		Mean	SD	Mean	SD
50 mg single dose	B, D	**3,153.56** ^*1^	809.71	**132.03** ^*2^	39.43
100 mg single dose	A, E, F	5,875.01	1,507.59	**251.77** ^*2^	79.85
100 mg/24 h for 6 days	C	6,449.18	1,590.68	**364.53** ^*2^	99.47

AUC_∞_ and C_max_ (single dose) are shown for A, B, D, E, F, and AUC_Ƭ_ and C_max_ (steady state) for C.

*^1^ *p* < 0.05 after ANOVA and Bonferroni *post-Hoc* vs. 100 mg single dose and 100 mg multiple dose.

*^2^ *p* < 0.05 after ANOVA and Bonferroni *post-Hoc* (all comparisons).

Females showed higher Cmax/DW than males (*p* = 0.040). Caucasians showed a higher tmax than the Mixed-race healthy volunteers (*p* = 0.007) (Table 4). Several differences in pharmacokinetic parameters were observed according to the clinical trial design, with clinical trial C (the only one with multiple-dose drug administration) showing the highest Cmax/DW and t1/2 and lowest tmax. Finally, a higher Cmax/DW was observed in fed healthy volunteers compared to fasting (Table 4).

**TABLE 4:** Pharmacokinetic parameters according to sex, race, clinical trial, and food administration conditions.

Variable		N	AUC/DW (kg*ng*h/mL*mg)		C_max_/DW (kg*ng/mL*mg)		t_max_ (h)		t_1/2_ (h)	
			Mean	SD	Mean	SD	Mean	SD	Mean	SD
Sex	Female	46	4,252.54	945.42	192.27*	**47.81**	6.56	1.64	10.52	3.97
	Male	52	4,146.35	1141.57	172.99	55.43	6.76	2.69	10.88	3.74
Race	Caucasian	26	4,413.79	1112.36	175.49	51.01	7.67*	**2.46**	10.42	2.38
	Mixed	72	4,117.63	1023.32	184.40	53.34	6.3	2.07	10.82	4.25
Cinical trial	A	18	3,634.07	959.35	163.05*^2^	**52.11**	6.89*^3^	**2.29**	8.89*^4^	**1.11**
	B	29	3,984.68	743.49	151.52*^2^	**33.31**	6.67*^3^	**2.41**	9.73*^4^	**1.85**
	C	9	4,486.00	1180.94	251.66*^2^	**61.86**	4.56*^3^	**1.81**	20.14*^4^	**5.12**
	D	19	4,815.53*^1^	**1267.14**	221.47*^2^	**23.56**	7.18*3	**1.63**	10.03*^4^	**1.99**
	E	17	4,365.82	947.12	161.24*^2^	**37.68**	7.15*^3^	**2.68**	10.52*^4^	**2.70**
	F	6	4,028.4	1071.16	216.10*^2^	**37.68**	6.08	0.20	9.52*4	**1.16**
Food	Fed	25	4,626.62	1249.39	220.18*	**26.79**	6.92	1.49	9.91	1.81
	Fasting	73	4,048.79	937.47	168.97	53.04	6.57	2.46	10.99	4.29
Total		98	4,196.20	1050.08	182,04	52.62	6.66	2.25	10.71	3.84

AUC refers to AUC∞ (single-dose) or AUCƬ (multiple-dose, clinical trial C). Cmax refers to Cmax (single-dose) or CmaxƬ (multiple-dose, clinical trial C).

*p < 0,05 after t-test.

* ^*1*^ *p < 0.05 after ANOVA and Bonferroni post-Hoc, D vs. A.*

* ^*2*^ *p < 0.05 after ANOVA and Bonferroni post-Hoc, A vs. C and D; B vs. C, D, and F; C vs. E; D vs. E.*

* ^*3*^ *p < 0.05 after ANOVA and Bonferroni post-Hoc, A vs. C; B vs. C; C vs. D and E.*

* ^4^ *p* < 0.05 after ANOVA and Bonferroni *post-Hoc*, A vs. C; B vs. C; C vs. D, E and F.

CYP2C19 RMs showed a higher Cmax/DW compared to NMs (*p* = 0.021) and UGT1A1 PMs showed a higher tmax compared to IMs + NMs (*p* = 0.034) (Table 5). However, none of these associations remained significant after Bonferroni correction for multiple comparisons. No other association was observed, including CYP2D6. The mean and SD of pharmacokinetic parameters according to the remaining genotypes or phenotypes is shown in the Supplementary Table S1. Due to the scarcity of associations, a multivariate analysis was not possible.

**TABLE 5:** Significant associations between CYP2C19 and UGT1A1 phenotypes and the pharmacokinetic parameters of desvenlafaxine.

Phenotype		AUC/DW (kg*ng*h/mL*mg)			C_max_/DW (kg*ng/mL*mg)			t_max_ (h)			t_1/2_ (h)		
		N	Mean	SD	N	Mean	SD	N	Mean	SD	N	Mean	SD
CYP2C19	UM	2	4,684.38	1284.89	2	198.67*	80.76	2	6.75	1.06	2	9.09	0.78
	RM	19	3,929.33	688.89	17	146.71*	**41.42**	19	7.74	2.79	19	10.67	4.86
	NM	56	4,248.93	1069.69	50	185.61	42.15	56	6.44	2.08	56	10.69	3.91
	IM	19	4,181.53	1263.15	18	164.99	50.31	19	6.37	2.17	19	10.97	2.9
	PM	2	4,906.20	1454.48	2	216.61	17.46	2	5.50	0.71	2	11.05	2.13
UGT1A1	NM	39	4,394.53	1140.16	34	175.80	43.44	39	6.86	2.05	39	11.53	4.41
	IM	48	4,017.38	998.73	44	173.38	48.43	48	6.19	2.17	48	10.17	3.57
	PM	11	4,273.31	868.63	11	178.99	51.53	11	8.05*1	**2.81**	11	10.16	2.18
Total		98	4,196.20	1050.08	89	175.00	46.46	98	6.66	2.25	98	10.71	3.84

*p < 0.05 vs. NM + IM + PM after t-test.

* ^1^ *p* < 0.05 vs. NM + IM after *t*-test.

AUC refers to AUC∞ (single-dose) or AUCƬ (multiple-dose, clinical trial C). Cmax refers to Cmax (single-dose) or CmaxƬ (multiple-dose, clinical trial C).

Concerning drug safety, the incidence of any ADRs was significantly higher in the only clinical trial with multiple-dose administration (clinical trial C) (*p* = 0.022) (Table 6). The incidence of the following individual ADRs was consistently higher in clinical trial C: xerostomia and taste disorders, dizziness, somnolence, other sleeping disorders and decreased appetite (Table 6). Volunteers experiencing any ADR showed a significantly higher AUC∞ than those not experiencing any ADR (5115.35 ± 1931.97 h*ng/mL vs. 4279.04 ± 1776.43 h*ng/mL respectively, *p* = 0.034), but similar Cmax. Furthermore, the incidence of asthenia was 20% in individuals with *ABCB1* rs203258 G/T genotype, 8.3% in those with A/A and 0% for those with G/G, G/A, T/T o A/T genotypes (*p* = 0.013). CYP2B6 RMs showed a higher incidence of asthenia (13% compared to 0% in NMs, IMs and PMs, *p* = 0.038). However, none of these associations remained significant after Bonferroni correction for multiple comparisons, therefore it was not possible to carry out the multivariate analysis.

**TABLE 6:** Incidence of ADRs according to clinical trial design.

*Clinical trial*	n	Any ADR	Xerostomia and taste disorders	Dizziness	Somnolence	Other sleeping disorders	Decreased appetite	Vomiting and nausea	Headache	Asthenia
*A*	18	38.90%	1 (5.6%)	2 (11.1%)	0 (0.0%)	0 (0.0%)	0 (0.0%)	3 (16.7%)	3 (16.7%)	2 (11.1%)
*B*	29	37.90%	0 (0.0%)	3 (10.3%)	4 (13.8%)	0 (0.0%)	0 (0.0%)	4 (13.8%)	5 (17.2%)	0 (0.0%)
*C*	9	77.80%	3 (33.3%)	4 (44.4%)	3 (33.3%)	3 (33.3%)	3 (33.3%)	0 (0.0%)	2 (22.2%)	0 (0.0%)
*D*	19	10.50%	0 (0.0%)	0 (0.0%)	0 (0.0%)	0 (0.0%)	0 (0.0%)	0 (0.0%)	2 (10.5%)	0 (0.0%)
*E*	17	47.10%	0 (0.0%)	2 (11.8%)	0 (0.0%)	0 (0.0%)	2 (11.8%)	4 (23.5%)	2 (11.8%)	1 (5.9%)
*F*	6	33.30%	0 (0.0%)	0 (0.0%)	0 (0.0%)	0 (0.0%)	0 (0.0%)	2 (33.3%)	1 (16.7%)	0 (0.0%)
*p value*		0.022	0.002	0.021	0.007	0.001	0.001	0.096	0.971	0.226

## Discussion

To date, numerous studies compared the efficacy and safety of antidepressants. In 2018, (Cipriani et al., 2018) published a comprehensive systematic review and meta-analysis of 21 antidepressant drugs and their use for the treatment of major depressive disorder. All antidepressants were found effective vs. placebo, with ORs ranging between 1.37 (for reboxetine) and 2.13 (for amitriptyline), with desvenlafaxine and venlafaxine showing intermediate effectiveness (OR = 1.49 and 1.78, respectively). In terms of dropout rate, both drugs showed a similar profile (ORs = 1.08 and 1.04 vs. placebo, respectively). Unfortunately, data comparing desvenlafaxine with other antidepressants is scarcely available, and placebo-controlled clinical trials are mainly available. With placebo as comparator, it seems that both drugs show a similar effectiveness profile, with desvenlafaxine showing a better safety profile (i.e., lower incidence of nausea) (Coleman et al., 2012). In a 8-week phase-III clinical trial, desvenlafaxine showed a 5% discontinuation rate compared to 6% for venlafaxine, and 13% for duloxetine (Tourian et al., 2009), which is consistent with desvenlafaxine having a slightly better safety profile. In summary, little evidence is available to justify the selection of other antidepressants in preference to desvenlafaxine. Otherwise, desvenlafaxine is one more drug in the therapeutic arsenal available to treat depression, and its selection over other antidepressant drugs depends on the clinician’s preference.

When comparing venlafaxine and desvenlafaxine, at equivalent doses, they could be considered similar therapeutic alternatives, as both compounds are potent serotonin and norepinephrine reuptake inhibitors. Venlafaxine, through CYP2D6, is converted to desvenlafaxine, hence, the pharmacological effects of desvenlafaxine can be expected when venlafaxine is administered; the only difference is that venlafaxine (the precursor) also inhibits dopamine reuptake and desvenlafaxine does not (Singh and Saadabadi, 2022). However, a clear drawback of venlafaxine therapy is the variability in response and safety associated with CYP2D6 phenotype. *A priori*, desvenlafaxine seems to have a better pharmacokinetic profile and a better dose-response and dose-safety relationship (Norman and Olver, 2021). In this work, not only did we confirm that the occurrence of ADRs caused by desvenlafaxine is associated with the time (multiple dose administration vs. single dose administration) and/or magnitude (AUC) of drug exposure, but we also clearly observed that the exposure to desvenlafaxine does not depend on the genetic polymorphism of candidate genes. The strong dose-dependent relationship with the occurrence of ADRs confirms that the mechanism of action of desvenlafaxine is essentially dose-dependent, compared to the biphasic dose-dependent mechanism of action of venlafaxine (Harvey et al., 2000). To our knowledge, this work is the first to analyze all potentially relevant pharmacogenes comprehensively and robustly with respect to the pharmacokinetics and safety of the drug. Although this might be somewhat obvious *a priori*, this does not hold for all drugs. For instance, voriconazole shows nonlinear pharmacokinetics, with the dose-response (or dose-safety) relationship exhibiting wide interpatient variability. This is typically explained by its limited capacity of elimination. Furthermore, CYP2C19 phenotype is a clear predictor of drug metabolism (Moriyama et al., 2017). Therefore, a reduction or increase in dose is not directly related to an increase in exposure or in the chances of achieving efficacy (Theuretzbacher et al., 2006; Zubiaur et al., 2021). Studies such as the present one are important to confirm this pharmacokinetic characteristic, which enables more predictable dosage adjustments of the drug.

Here, the healthy volunteers presented pharmacokinetic parameters consistent with previous literature. For instance, an AUC_∞_ of 5875.01 ng*h/mL was observed for the 100 mg formulation, compared to 6747 ng*h/mL and a Cmax of 251 ng/mL compared to 376 ng/mL (Spanish Drug Agency, 2012) (Spanish Drug Agency, 2012). Furthermore, the global tmax was 6.66 h compared to 7.5 h, and a t1/2 of 10.71 compared to 11 h (Spanish Drug Agency, 2012). Female healthy volunteers presented a higher Cmax/DW, which may be explained by sex-specific physiological differences in drug absorption (Soldin and Mattison, 2009). A clear dose-dependent relationship was observed in the variations of AUC and Cmax. Furthermore, several differences in pharmacokinetic variables were observed according to the clinical trial design. However, these differences were small, with the AUC/DW ranging from 86% to 115% with respect to mean AUC/DW, and the Cmax/DW ranging from 83% to 121%, except for the clinical trial C, which showed a 138% with respect to mean C_max_/DW. This difference can be explained by the fact that, in clinical trial C, several desvenlafaxine doses were administered and the residual concentration from previous drug administrations needs to be considered. In contrast, AUC∞ from single dose clinical trials and AUCƬ from the multiple-dose clinical trial were equivalent, as expected. The remaining differences related to the clinical trial design can be considered spurious and related to the low sample size for each clinical trial. Finally, fed healthy volunteers showed a higher C_max_/DW, an effect that is well described in desvenlafaxine’s drug label (Spanish Drug Agency, 2012).

As mentioned earlier, the incidence of ADRs was significantly higher in the only clinical trial with multiple-dose administration (clinical trial C). This may be explained by the higher observed Cmax/DW or the prolonged exposure to the drug. Furthermore, the AUC was identified as the best predictor of ADR development; the fact that drug exposure is the main predictor of drug tolerability is reassuring. Assuming linear pharmacokinetics, ADRs resulting from drug overexposure could be resolved with dose adjustments. This does not apply equally to venlafaxine, as certain patients (e.g., CYP2D6 UMs or PMs) will respond variably to similar adjustments. The association between CYP2B6 RMs and asthenia incidence can be considered spurious; should CYP2B6 metabolize desvenlafaxine, a lower rate of ADRs should be expected in individuals with a higher metabolic capacity. Alternatively, this would be explained if desvenlafaxine had an active metabolite responsible for this side effect. However, to our knowledge, this metabolite has not been described to date. Likewise, the association between *ABCB1* rs203258 G/T and asthenia can only be explained as a spurious event because a heterozygous disadvantage is not possible for traits inherited in a codominant manner.

Several study limitations should be considered. The clinical trial study design did not allow to collect data on the long-term pharmacokinetics and tolerability or effectiveness. We were also unable to analyze the influence of Black race on desvenlafaxine pharmacokinetics because there was only one subject self-reported as Black. In addition, some Tier 1 variants in *CYP2C9* and *CYP2D6* were not included (Pratt et al., 2019; Pratt et al., 2021) thus the pharmacogenetic phenotype may not have been correctly inferred, albeit this possibility is very unlikely due to the low prevalence of these alleles (e.g., CYP2C9*8). In addition, among the UGT enzymes, only variants in *UGT1A1* have been demonstrated to alter the enzyme’s function, while no variants with a functional impact are described for other important *UGT* candidates such as *UGT1A3* or *UGT2B4*. Furthermore, these results may be interpreted with caution given the limited sample size, which implies that the statistical power is limited and may not be sufficient to detect pharmacogenetic effects, especially for rare polymorphisms. In addition, some associations are described that did not reach the significance level after Bonferroni correction for multiple comparisons, but these associations must be considered cautiously due to the exploratory nature of this work. In contrast, bioequivalence clinical trials offer a controlled setting for the evaluation of pharmacokinetic variability based on genetic polymorphism or demographics as confounding factors are avoided.

## Conclusion

Desvenlafaxine pharmacokinetic parameters were unrelated to genetic polymorphism of the principal candidate pharmacogenes. In contrast, drug exposure in time and magnitude (AUC) were the principal predictors of ADR incidence, which confirms that the mechanism of action of desvenlafaxine is essentially dose-dependent. These two findings can be interpreted as therapeutic advantages of desvenlafaxine compared to venlafaxine.

## Data Availability

The original contributions presented in the study are included in the article/Supplementary Material, further inquiries can be directed to the corresponding authors.
