# Supplementary material for: Impact of polymorphisms in CYP and UGT enzymes and ABC and SLCO1B1 transporters on the pharmacokinetics and safety of desvenlafaxine
Source: Front Pharmacol. 2023 Feb 2;14:1110460. doi: 10.3389/fphar.2023.1110460 (PMC9934922; doi:10.3389/fphar.2023.1110460)
Supplement: Supplementary file 1 [file Table1.DOCX]

Supplementary Material

Impact of polymorphisms in CYP and UGT enzymes and ABC and SLCO1B1 transporters on the pharmacokinetics and safety of desvenlafaxine.

**Sofía Calleja^1^, Pablo Zubiaur^1,2*^, Dolores Ochoa^1^, Gonzalo Villapalos-García^1^, Gina Mejia-Abril^1^, Paula Soria-Chacartegui^1^, Marcos Navares-Gómez^1^, Alejandro de Miguel^1^, Manuel Román^1^, Samuel Martín-Vílchez^1^, Francisco Abad-Santos ^1,3*^.**

^1^Clinical Pharmacology Department, Hospital Universitario de La Princesa, Instituto Teófilo Hernando, Universidad Autónoma de Madrid (UAM), Instituto de Investigación Sanitaria La Princesa (IP), Madrid, Spain.

^2^Division of Clinical Pharmacology, Toxicology and Therapeutic Innovation, Children's Mercy Research Institute, Kansas City, MO, United States.

^3^Centro de Investigación Biomédica en Red de Enfermedades Hepáticas y Digestivas (CIBERehd), Instituto de Salud Carlos III, Madrid, Spain.

*** Correspondence:**Corresponding Authors

[pablo.zubiaur@uam.es](mailto:pablo.zubiaur@uam.es); [francisco.abad@uam.es](mailto:francisco.abad@uam.es)

Supplementary Table 1. Description of pharmacokinetic parameters according to genotypes or phenotypes not showing a significant relationship.

| Genotype or phenotype | | AUC/DW (kg*ng*h/mL*mg) | | | C_max_/DW (kg*ng/mL*mg) | | | t_max_ (h) | | | t_1/2_ (h) | | |
| --- | --- | --- | --- | --- | --- | --- | --- | --- | --- | --- | --- | --- | --- |
|  |  | N | Mean | SD | N | Mean | SD | N | Mean | SD | N | Mean | SD |
| ABCB1 C1236T rs1128503 | C/C | 39 | 4183.59 | 1098.45 | 35 | 187.57 | 51.94 | 39 | 6.32 | 2.35 | 39 | 10.52 | 3.91 |
|  | C/T | 41 | 4131.94 | 858.35 | 37 | 179.77 | 55.93 | 41 | 7.04 | 2.46 | 41 | 10.60 | 3.42 |
|  | T/T | 15 | 4415.25 | 1472.02 | 14 | 176.55 | 50.31 | 15 | 6.77 | 1.29 | 15 | 11.64 | 5.06 |
| ABCB1 C3435T rs1045642 | C/C | 35 | 4269.69 | 984.59 | 32 | 189.27 | 49.47 | 35 | 6.33 | 2.44 | 35 | 10.36 | 3.83 |
|  | C/T | 44 | 4100.39 | 989.86 | 40 | 184.04 | 56.68 | 44 | 7.10 | 2.23 | 44 | 10.24 | 3.03 |
|  | T/T | 19 | 4282.69 | 1315.10 | 17 | 164.06 | 46.60 | 19 | 6.26 | 1.81 | 19 | 12.46 | 5.07 |
| ABCB1 G2677A/T rs203258 | G/G | 32 | 4130.31 | 1120.73 | 28 | 186.85 | 46.89 | 32 | 6.06 | 2.41 | 32 | 10.53 | 4.38 |
|  | G/A | 39 | 4082.80 | 872.38 | 37 | 171.55 | 45.10 | 39 | 7.18 | 2.40 | 39 | 10.48 | 3.17 |
|  | G/T | 10 | 4502.10 | 836.23 | 8 | 212.45 | 45.33 | 10 | 6.60 | 2.18 | 10 | 10.67 | 2.74 |
|  | A/A | 12 | 4623.57 | 1529.83 | 11 | 180.50 | 52.33 | 12 | 6.54 | 1.41 | 12 | 12.50 | 5.40 |
|  | T/T+A/T | 4 | 3572.03 | 586.62 | 4 | 187.16 | 33.61 | 4 | 6.50 | 1.00 | 4 | 8.67 | 0.63 |
| ABCG2 rs2231142 | NF | 73 | 4143.72 | 932.62 | 67 | 172.32 | 46.29 | 73 | 6.79 | 2.47 | 73 | 10.70 | 3.94 |
|  | DF | 23 | 4335.35 | 1404.89 | 20 | 187.03 | 47.73 | 23 | 6.28 | 1.40 | 23 | 10.81 | 3.76 |
|  | PF | 2 | 4511.56 | 260.62 | 2 | 144.54 | 6.18 | 2 | 6.50 | 2.12 | 2 | 9.87 | 0.08 |
| CYP2B6 | RM | 23 | 4033.15 | 1145.54 | 22 | 165.93 | 47.65 | 23 | 6.87 | 2.70 | 23 | 9.48 | 2.00 |
|  | NM | 32 | 4225.26 | 1183.10 | 30 | 179.00 | 45.68 | 32 | 6.52 | 1.49 | 32 | 10.66 | 4.42 |
|  | IM | 33 | 4348.13 | 853.29 | 30 | 176.59 | 44.73 | 33 | 6.46 | 1.94 | 33 | 10.96 | 2.89 |
|  | PM | 10 | 3976.84 | 1028.33 | 7 | 179.49 | 59.51 | 10 | 7.35 | 3.87 | 10 | 12.88 | 6.52 |
| CYP2C8 | UM | 1 | 4207.26 | 0.00 | 1 | 132.30 | 0.00 | 1 | 7.50 | 0.00 | 1 | 13.08 | 0.00 |
|  | RM | 20 | 4217.51 | 973.25 | 17 | 174.76 | 41.59 | 20 | 7.25 | 2.85 | 20 | 10.53 | 3.45 |
|  | NM | 68 | 4136.08 | 914.48 | 62 | 176.98 | 44.93 | 68 | 6.50 | 2.00 | 68 | 10.76 | 4.14 |
|  | IM | 9 | 4601.87 | 1974.70 | 9 | 166.54 | 67.50 | 9 | 6.50 | 2.73 | 9 | 10.51 | 2.43 |
| CYP2C9 | NM | 69 | 4070.87 | 907.46 | 63 | 173.13 | 47.46 | 69 | 6.41 | 2.05 | 69 | 10.58 | 4.04 |
|  | IM | 27 | 4374.28 | 1150.59 | 24 | 173.50 | 39.55 | 27 | 7.26 | 2.70 | 27 | 10.91 | 3.33 |
|  | PM | 2 | 6115.92 | 2689.08 | 2 | 251.83 | 47.58 | 2 | 7.50 | 1.41 | 2 | 12.79 | 3.89 |
| CYP2D6 | UM | 7 | 4516.42 | 945.43 | 7 | 170.35 | 44.42 | 7 | 7.64 | 1.21 | 7 | 10.00 | 1.53 |
|  | NM | 69 | 4217.37 | 942.63 | 60 | 173.96 | 43.10 | 69 | 6.67 | 2.42 | 69 | 11.1 | 4.38 |
|  | IM | 17 | 3777.06 | 1167.66 | 17 | 168.84 | 52.77 | 17 | 6.12 | 1.83 | 17 | 9.59 | 1.52 |
|  | PM | 4 | 5251.06 | 1874.23 | 4 | 220.58 | 69.53 | 4 | 7.64 | 1.96 | 4 | 10.32 | 3.48 |
| CYP3A4 | NM | 94 | 4217.43 | 1063.52 | 85 | 173.78 | 46.88 | 94 | 6.68 | 2.29 | 94 | 10.78 | 3.90 |
|  | IM | 4 | 3697.32 | 493.57 | 4 | 200.78 | 29.48 | 4 | 6.25 | 1.26 | 4 | 9.19 | 0.45 |
| CYP3A5 | NM | 3 | 4360.32 | 407.26 | 3 | 186.49 | 50.28 | 3 | 6.33 | 1.26 | 3 | 8.61 | 1.18 |
|  | IM | 27 | 4125.50 | 823.00 | 24 | 173.20 | 41.89 | 27 | 6.83 | 2.59 | 27 | 10.95 | 3.99 |
|  | PM | 68 | 4217.03 | 1151.07 | 62 | 175.14 | 48.60 | 68 | 6.61 | 2.16 | 68 | 10.71 | 3.86 |
| SLCO1B1 | NM | 70 | 4166.41 | 1029.08 | 64 | 178.39 | 47.83 | 70 | 6.39 | 2.15 | 70 | 10.51 | 3.53 |
|  | IM | 26 | 4325.70 | 1139.72 | 23 | 170.93 | 40.73 | 26 | 7.06 | 1.74 | 26 | 11.39 | 4.67 |
|  | PM | 2 | 3555.15 | 307.47 | 2 | 113.32 | 24.86 | 2 | 11.00 | 7.07 | 2 | 9.08 | 0.16 |
| UGT2B15 | A/A | 24 | 4517.41 | 1314.04 | 19 | 180.52 | 51.82 | 24 | 7.06 | 3.30 | 24 | 11.64 | 3.90 |
|  | A/C | 38 | 4169.46 | 1025.52 | 35 | 173.35 | 47.14 | 38 | 6.57 | 1.90 | 38 | 10.76 | 3.71 |
|  | C/C | 35 | 4189.91 | 838.99 | 34 | 171.99 | 43.37 | 35 | 6.52 | 1.73 | 35 | 10.08 | 3.94 |
| Total | | 98 | 4196.20 | 1050.08 | 89 | 175.0 | 46.46 | 98 | 6.66 | 2.25 | 98 | 10.71 | 3.84 |
